# Late Arc/Arg3.1 expression in the basolateral amygdala is essential for persistence of newly-acquired and reactivated contextual fear memories

**DOI:** 10.1038/srep21007

**Published:** 2016-02-16

**Authors:** Daisuke Nakayama, Yoshiko Hashikawa-Yamasaki, Yuji Ikegaya, Norio Matsuki, Hiroshi Nomura

**Affiliations:** 1 Laboratory of Chemical Pharmacology, Graduate School of Pharmaceutical Sciences, The University of Tokyo, Tokyo, Japan; 2 Center for Information and Neural Networks, Suita City, Osaka, Japan

## Abstract

A feature of fear memory is its persistence, which could be a factor for affective disorders. Memory retrieval destabilizes consolidated memories, and then rapid molecular cascades contribute to early stabilization of reactivated memories. However, persistence of reactivated memories has been poorly understood. Here, we discover that late Arc (also known as Arg3.1) expression in the mouse basolateral amygdala (BLA) is involved in persistence of newly-acquired and reactivated fear memories. After both fear learning and retrieval, Arc levels increased at 2 h, returned to basal levels at 6 h but increased again at 12 h. Inhibiting late Arc expression impaired memory retention 7 d, but not 2 d, after fear learning and retrieval. Moreover, blockade of NR2B-containing N-methyl-D-aspartate receptors (NMDARs) prevented memory destabilization and inhibited late Arc expression. These findings indicate that NR2B-NMDAR and late Arc expression plays a critical role in the destabilization and persistence of reactivated memories.

A persistent fear memory could be a factor for affective disorders. For example, the characteristics of post-traumatic disorder (PTSD) is that the patients suffer flashbacks or avoidance of memories of traumatic events for more than a month after the events occurred. Erasing the memories of traumatic events is expected to be a novel approach to treating PTSD^1^. Although early memory formation is built on rapid molecular cascades within a brief time window after learning^2^,^3^,^4^,^5^,^6^,^7^, the mechanisms for memory persistence are less understood. A few recent studies have found that inhibition of brain-derived neurotrophic factor (BDNF) or c-Fos at 12–24 h after learning impairs memory retention at 7 d but not 2 d after learning^8^,^9^,^10^. They have proposed that the delayed biochemical changes are possible regulators of memory persistence.

Reactivation of memories destabilizes once consolidated memories, and their early stabilization is based on *de novo* gene expression^11^,^12^,^13^. It is possible that persistence of reactivated memories, as well as new memories, requires additional cellular signaling. In fact, we have previously shown that inhibition of protein synthesis at 12 h after memory retrieval impaired memory retention at 7 d but not 2 d after memory retrieval^14^. However, the precise molecular mechanisms for persistence of reactivated memories are unclear.

*Arc* (also known as *Arg3.1*) is an immediate early gene that is required for the consolidation of synaptic plasticity and long-term memory^15^,^16^,^17^. Arc expression is rapidly upregulated following both initial learning and memory retrieval, and is required for early stabilization of newly acquired and reactivated memories^15^,^16^,^17^,^18^,^19^. We previously found that late-phase Arc expression in the hippocampus after learning is involved in the persistence of newly-acquired memories^20^. Thus, we hypothesized that late phase of Arc expression contributes to persistence of reactivated memories as well as newly acquired memories. In this study, we characterized Arc expression in the basolateral amygdala (BLA) after contextual fear conditioning and fear memory retrieval, and revealed the role of late Arc expression in newly acquired and reactivated memories. Then, we investigated the mechanisms that prime late Arc expression.

## Results

### Early and late Arc expression after contextual fear learning and retrieval

**Figure 1:**
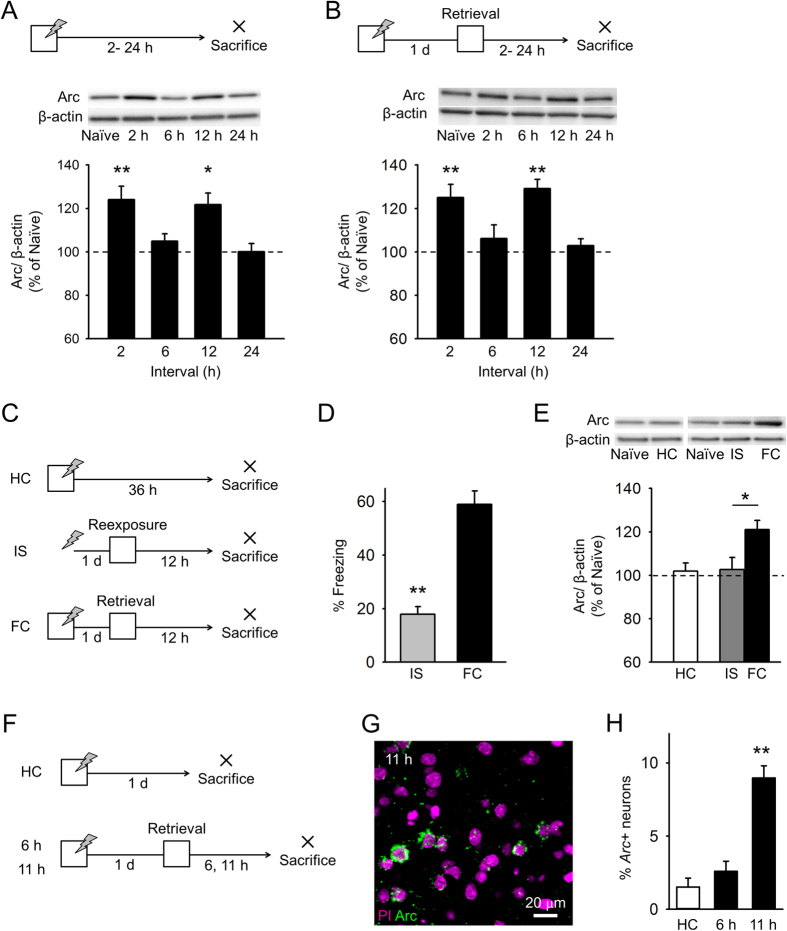
Early and late Arc expression following contextual fear learning and retrieval (**A**) Arc levels in the BLA increased 2 and 12 h after learning, but not 6 and 24 h after learning, compared to control levels, n = 10 mice per group. (**B**) Arc levels increased 2 and 12 h after context re-exposure, but not 6 and 24 h after, compared to control levels. n = 10 mice per group. (**C**) The experimental procedure for (**D,E**) (HC, n = 10 mice; IS, n = 13 mice; FC, n = 13 mice). (**D**) The IS group exhibited less freezing behavior than the FC group. (**E**) BLA Arc levels in the HC group were comparable to those in naïve mice (n = 10 mice). BLA Arc levels in the IS group were comparable to those in naïve mice (n = 13 mice) and were lower than those in the FC group. (**F**) The experimental procedure for (**G,H**) (HC, n = 6 mice; 6 h, n = 3 mice; 11 h, n = 6 mice). (**G**) Representative image of *Arc* RNA expression in the BLA 11 h after fear memory retrieval. (**H**) The proportion of *Arc* + neurons in the BLA increased 12 h after fear memory retrieval. **P < 0.01, *P < 0.05.

We measured BLA Arc levels following contextual fear conditioning. Mice were sacrificed 2, 6, 12, and 24 h after fear conditioning, and BLA lysates were subjected to western blotting with an anti-Arc antibody (Fig. 1A). Compared to those of naïve control mice, following fear conditioning, BLA Arc levels increased at 2 h, returned to basal levels at 6 h, increased again at 12 h, and returned to basal levels at 24 h (Fig. 1A).

Next, we characterized Arc expression after fear memory retrieval. We subjected mice to contextual fear conditioning on day 1 and re-exposure to a conditioned context on day 2 (Fig. 1B). Mice exhibited freezing behavior upon exposure to the conditioned context (freezing time; 2 h, 63.4 ± 4.9%; 6 h, 57.5 ± 4.3%; 12 h, 59.4 ± 2.5%; 24 h, 59.0 ± 3.2%), indicating that they recalled a fear memory. Mice were sacrificed 2, 6, 12, or 24 h after re-exposure. Compared to those of naïve control mice, following re-exposure, BLA Arc levels increased at 2 h, returned to basal levels at 6 h, increased again at 12 h, and returned to basal levels at 24 h (Fig. 1B). When mice received fear conditioning and were sacrificed 36 h later without re-exposure to the conditioned context (Fig. 1C, homecage (HC) group), Arc levels at this time point were comparable to basal levels (Fig. 1E). In addition, Arc expression was measured in mice subjected to an immediate shock session instead of a fear conditioning session (Fig. 1C, IS group). The immediate shock protocol temporally dissociated the context and shock exposure in mice and resulted in poor memory retention^21^. Freezing time of mice receiving the immediate shock was shorter than that of mice given standard fear conditioning (Fig. 1D), although it was still longer than the freezing time reported in previous studies^22^ possibly because the overall duration of shock was longer (6 s). Arc levels 12 h after re-exposure to the context were comparable to basal levels (Fig. 1E). These data indicate that late Arc expression depends on retrieval of an associative fear memory.

In addition, we investigated whether late Arc protein expression (at 12 h) was accompanied by an increase in *Arc* transcription. We prepared different mice and sacrificed 6 or 11 h after re-exposure to the conditioned context (Fig. 1F). Brain slices, including the BLA, were subjected to fluorescent *in situ* hybridization (Fig. 1G). We measured the proportion of neurons expressing cytoplasmic *Arc*, which is detected for longer time than nuclear *Arc*. The proportion of *Arc*+ neurons in the BLA at 11 h, but not at 6 h, was greater than that at basal levels (Fig. 1H). These results suggest that fear memory retrieval induces late Arc expression in the BLA through increased transcription.

### Late Arc expression contributes to the persistence of newly-acquired fear memory

**Figure 2:**
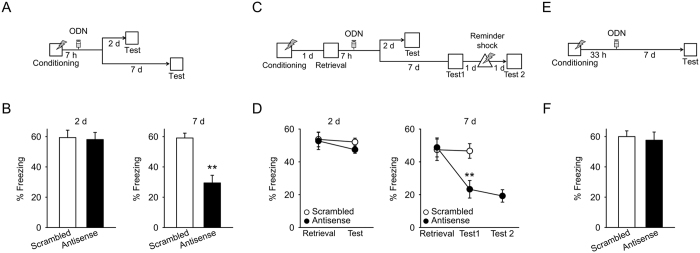
Late Arc expression contributes to persistence of newly-acquired and reactivated fear memories. (**A**) The experimental procedure for (**B**). (**B**) (Left) *Arc* antisense ODN treatment 7 h after conditioning did not affect freezing behavior in the 2 d test (n = 9 mice). (Right) *Arc* antisense ODN treatment 7 h after conditioning disrupted freezing behavior in the 7 d test (n = 8, 9 mice). (**C)** The experimental procedure for (**D**). (**D**) (Left) *Arc* antisense ODN treatment 7 h after re-exposure had no effect on freezing behavior in the 2 d test (n = 8 mice). (Right) *Arc* antisense ODN treatment 7 h after re-exposure impaired freezing behavior in the 7 d test (n = 7 mice). The reminder shock did not affect freezing behavior in a subsequent test. (**E**) The experimental procedure for (**F**). (**F**) *Arc* antisense ODN treatment in the absence of memory retrieval did not affect freezing behavior in the 7 d test (n = 7 mice). Histological verification of cannula placements are shown in supplementary Fig. S1. **P < 0.01, *P < 0.05.

To investigate the effects of late Arc expression on newly-acquired fear memories, we employed *Arc* antisense oligodeoxynucleotides (ODNs). We previously confirmed that infusion of an *Arc* antisense ODN (400 pmol/hemisphere) into the dorsal hippocampus inhibits hippocampal Arc expression (5 h after infusion) and has no effect on the expression of other immediate-early genes^23^. We also confirmed that infusion of an *Arc* antisense ODN inhibited late Arc expression following memory retrieval in the BLA (Supplementary Fig. S1). Then, we infused *Arc* antisense or scrambled ODNs into the BLA 7 h after fear conditioning (Supplementary Fig. S2), and 2 d later, mice were placed into the conditioned context to test memory retention (Fig. 2A). In contrast to a previous study reporting that inhibition of early Arc expression impairs freezing behavior in a test performed 1 d after infusion of an antisense ODN^15^, inhibition of late Arc expression did not affect freezing behavior 2 d after infusion (Fig. 2B, left). Next, we prepared different mice and tested memory retention 7 d later, instead of 2 d after receiving *Arc* antisense or scrambled ODN infusions (Fig. 2A). Inhibition of late Arc expression disrupted freezing behavior in the 7 d test (Fig. 2B, right). These results indicate that late Arc expression in the BLA contributes to the persistence but not formation of newly-acquired memories.

### Late Arc expression contributes to the persistence of reactivated fear memory

To test whether late Arc expression after memory retrieval, as well as after initial learning, contributes to memory persistence, we infused *Arc* antisense or scrambled ODN into the BLA 7 h after re-exposure to the conditioned context (Fig. 2C). Memory retention was tested either 2 d or 7 d later. Inhibition of late Arc expression had no effect on freezing behavior during the 2 d test but disrupted freezing behavior in the 7 d test (Fig. 2D). This result suggests that late Arc expression after memory retrieval contributes to memory persistence. However, it is possible that decrease in freezing behavior observed after inhibition of late Arc expression was the result of increased fear extinction rather than memory impairment. If this was the case, a weak immediate shock (reminder shock) would reinstate conditioned fear^24^,^45^. To test this possibility, we gave a reminder shock to mice receiving the *Arc* antisense ODN 1 d after testing memory retention, and examined memory retention again 1 d later (Fig. 2C). The reminder shock did not increase freezing behavior (Fig. 2D, right), suggesting that the observed decrease in freezing behavior was not due to increased fear extinction. Furthermore, we tested whether the memory impairment resulting from infusion of *Arc* antisense ODN depended on memory retrieval. Mice were infused with *Arc* antisense or scrambled ODNs into the BLA 31 h after fear conditioning (without memory retrieval), and memory retention was tested 7 d later (Fig. 2E). Infusions of *Arc* antisense ODN in the absence of memory retrieval did not affect freezing behavior in the 7 d test (Fig. 2F). These results indicate that late Arc expression following memory retrieval contributes to the persistence of reactivated memories.

### NR2B-NMDARs are involved in memory destabilization that entails early and late Arc expression

Our finding that inhibition of late Arc expression impaired retention of reactivated memories 7 d after treatment suggests that reactivated memories are destabilized and then stabilized in a late Arc expression-dependent manner. However, the destabilization process that entails late Arc expression is unclear. Previous studies have shown that inhibition of NR2B-containing N-methyl-D-aspartate receptors (NMDARs) prevents memory impairment resulting from inhibition of protein synthesis following memory reactivation^26^,^27^. The studies suggested that activation of NR2B-NMDARs is involved in memory destabilization that entails early protein synthesis after memory reactivation. Thus, NR2B-NMDAR activation could be involved in memory destabilization that entails late Arc expression, as well as early Arc expression.

First, we tested whether blockade of NR2B-NMDARs prevents memory impairment resulting from inhibiting protein synthesis in our experimental conditions. We infused ifenprodil, an NR2B-NMDAR antagonist, or vehicle into the BLA 5 min before memory retrieval, and also infused anisomycin, a protein synthesis inhibitor, immediately after memory retrieval (Supplementary Fig. S3a). Consistent with previous studies, blockade of NR2B-NMDARs prevented memory impairment resulting from inhibition of early protein synthesis following memory reactivation (Supplementary Fig. S3b). We also prepared different mice and infused ifenprodil or vehicle into the BLA before memory retrieval and anisomycin 10 h after memory retrieval. Infusions of ifenprodil prevented memory impairment resulting from inhibition of late protein synthesis (Supplementary Fig. S3c,d).

**Figure 3:**
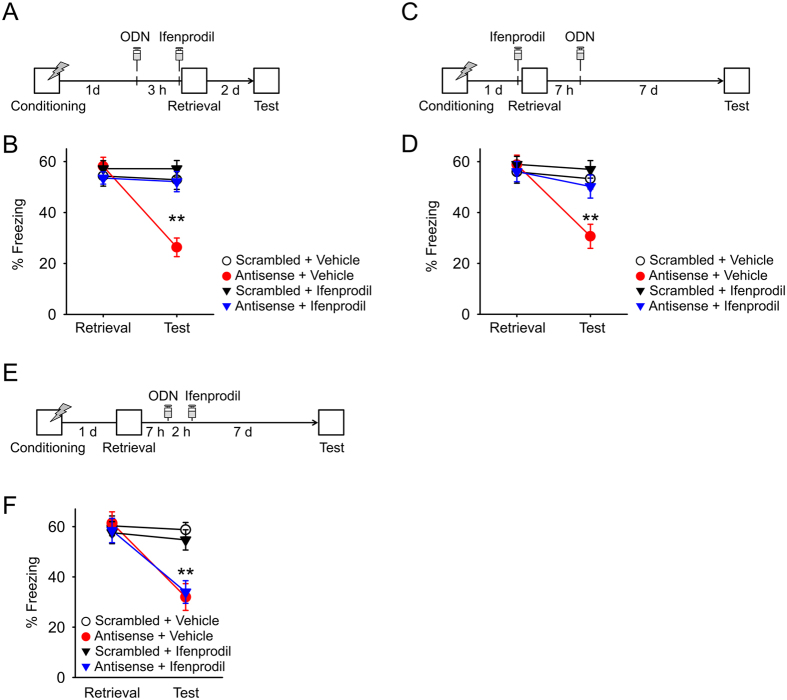
NR2B-NMDARs are involved in memory destabilization that entails early and late Arc expression (**A**) The experimental procedure for (**B**) (scrambled + Vehicle: n = 11 mice, antisense + Vehicle: n = 12 mice, scrambled + Ifenprodil: n = 11 mice, antisense + Ifenprodil: n = 12 mice). (**B**) *Arc* antisense ODN treatment disrupted freezing behavior 2 d after memory retrieval. Combinatorial infusions of ifenprodil prevented this memory impairment. (**C**) The experimental procedure for (**D**) (scrambled + Vehicle: n = 11 mice, antisense + Vehicle: n = 11 mice, scrambled + Ifenprodil: n = 11 mice, antisense + Ifenprodil: n = 12 mice). (**D**) Infusions of *Arc* antisense ODNs disrupted freezing behavior 7 d after memory retrieval. Combinatorial infusions of ifenprodil prevented this memory impairment. (**E)** The experimental procedure for (**F**) (Scrambled + Vehicle: n = 11 mice, Antisense + Vehicle: n = 11 mice, Scrambled + Ifenprodil: n = 12 mice, Antisense + Ifenprodil: n = 12 mice). (**F**) Infusions of *Arc* antisense ODN disrupted freezing behavior 7 d after memory retrieval. Combinatorial infusions of ifenprodil did not affect this memory impairment. Histological verification of cannula placements are shown in supplementary Fig. S3. **P < 0.01, *P < 0.05.

Next, we determined whether blockade of NR2B-NDMARs prevents memory impairment resulting from inhibition of early Arc expression. We infused *Arc* antisense or scrambled ODNs into the BLA 3 h before memory retrieval, and also infused ifenprodil or vehicle 5 min before memory retrieval (Fig. 3A). Memory retention was tested 2 d later. While inhibition of early Arc expression impaired freezing behavior in the 2 d test (Fig. 3B; Antisense + Vehicle group), this impairment was prevented by a combinatorial infusion of ifenprodil (Antisense + Ifenprodil). Infusions of ifenprodil alone did not affect freezing behavior in this test (Scrambled + Ifenprodil group). All behavioral groups showed comparable freezing behavior in a retrieval session (Fig. 3B). These results suggest that NR2B-NMDAR activation is involved in memory destabilization that entails early Arc expression.

To test whether blockade of NR2B-NDMAR prevents memory impairment resulting from inhibition of late Arc expression, we infused ifenprodil or vehicle into the BLA 5 min before memory retrieval and also infused *Arc* antisense or scrambled ODNs 7 h after memory retrieval (Fig. 3C). Memory retention was tested 7 d later. Consistent with the previous result (Fig. 2D), inhibition of late Arc expression impaired freezing behavior in the 7 d test (Fig. 3D; Antisense + Vehicle group). However, combinatorial infusions of ifenprodil prevented this impairment (Antisense + Ifenprodil). Infusions of ifenprodil alone did not affect freezing behavior (Scrambled + Ifenprodil group).

When mice received infusions of ifenprodil 9 h after retrieval, ifenprodil infusions did not prevent memory impairment resulting from inhibition of late Arc expression (Fig. 3E,F). These results indicate that activation of NR2B-NMDARs at the time of memory retrieval is involved in memory destabilization that entails late Arc expression.

Milton *et al.* showed that NR2A-NMDARs are involved in restabilization of cued fear memory^26^. We asked whether restabilization of contextual fear memory requires NR2A-NMDARs activation. Mice were given infusions of NVP-AAM077, NR2A-preferring NMDAR antagonist, into the BLA 5 min before memory retrieval (Supplementary Fig. S5a). NVP-AAM077 infusions disrupted freezing behavior at the test, indicating that NR2A-NMDARs are involved in memory restabilization of contextual fear memory.

### NR2B-NMDARs are essential for early and late Arc expression

**Figure 4:**
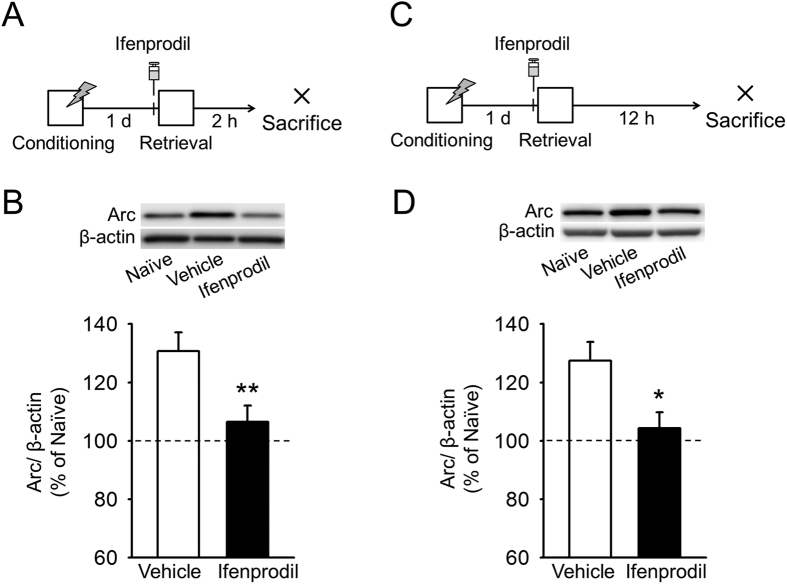
Early and late Arc expression after memory retrieval depend on activation of NR2B-NMDARs. (**A**) The experimental procedure for (**B**) (n = 8 mice for each group). (**B**) Ifenprodil infusions inhibited Arc expression at the 2 h time point. (**C**) The experimental procedure for (**D**) (n = 8 mice for each group). (**D**) Ifenprodil infusions inhibited Arc expression at the 12 h time point. Histological verification of cannula placements are shown in supplementary Fig. S6. **P < 0.01, *P < 0.05.

Finally, we investigated whether early and late Arc expression depends on NR2B-NMDAR activation. Mice received infusions of ifenprodil or vehicle into the BLA 5 min before memory retrieval and were sacrificed either 2 h or 12 h later (Fig. 4A,C). Consistent with the previous results (Fig. 1D), Arc levels in the BLA increased at 2 h and 12 h in vehicle-treated mice (Fig. 4B,D). In ifenprodil-treated mice, however, Arc levels at 2 h and 12 h were comparable to basal levels (Fig. 4B,D). These results indicate that NR2B-NMDAR activation primes early and late Arc expression, which contributes to early and late memory stabilization.

## Discussion

In the present study, we found that late Arc expression following fear learning and retrieval is essential for persistence of newly-acquired and reactivated fear memories, respectively. We also found that blockade of NR2B-NMDARs prior to memory retrieval prevented memory destabilization that entails early and late Arc expression and that this blockade also inhibited early and late Arc expression. Therefore, NR2B-NMDAR signaling is likely to be involved in both destabilization and restabilization of reactivated fear memories.

Similarities between the stabilization of newly-acquired memories (consolidation) and restabilization of reactivated memories (reconsolidation) are under debate^2^,^12^. Although memory consolidation and reconsolidation utilize many common mechanisms^2^,^12^, some studies reported that distinct molecular cascades and brain regions are involved in consolidation and reconsolidation^12^,^28^,^29^. In our current study, unlike previous studies, we focused on persistence of newly-acquired and reactivated memories. We found that BLA Arc levels were upregulated 12 h after initial learning and 12 h after memory retrieval and that late Arc expression is required for persistence of both new and reactivated memories. Therefore, persistence of newly-acquired and reactivated memories may require, at least in part, common molecular mechanisms.

It is difficult to exactly separate processes involved in persistence and formation of newly-acquired memories. Using antisense ODN approach, we found that early and late Arc expression following fear conditioning are required for formation and persistence of fear memory, respectively. However, early alterations in Arc expression could be reflective both of conditioning (e.g. context-shock convergence) itself and of memory formation. Therefore, early Arc expression might also contribute to the conditioning itself.

NR2B-NMDAR signaling is involved in destabilization of reactivated memories. A previous study found that ifenprodil infusions prevented memory impairments induced by anisomycin, a protein synthesis inhibitor^26^,^27^. In the present study, ifenprodil prevented memory impairment resulting from inhibition of early Arc expression following memory retrieval. This result indicates that reactivated memories that are destabilized through NR2B-NMDARs are restabilized through early Arc expression. Furthermore, we found that ifenprodil infusions administered prior to memory retrieval prevented memory impairment resulting from inhibition of late Arc expression. These findings indicate that activation of NR2B-NMDARs at the time of memory retrieval triggers memory destabilization that entails both early memory stabilization and memory persistence. NR2B-NMDARs strongly interact with Ca^2+^/calmodulin-dependent protein kinase II (CaMKII), which recruits proteasomes to dendritic spines^30^,^31^. Thus, activation of NR2B-NMDA receptors may induce protein degradation, which leads to memory destabilization^32^.

NMDAR signaling is essential for Arc expression following memory retrieval, which is implicated in memory restabilization. A previous study demonstrated that although NR2A-NMDAR signaling is involved in restabilization but not destabilization of reactivated memories, NR2B-NMDAR signaling is involved in memory destabilization^26^. However, it was not determined whether activation of NR2B-NMDARs is important for memory restabilization. In our study, ifenprodil infusions inhibited early and late Arc expression following memory retrieval, which are implicated in early and late stabilization of reactivated memories, respectively. These results suggest that NR2B-NMDA receptor signaling is involved in the restabilization of reactivated memories as well as their destabilization. Ifenprodil infusions had no effect on memory retention, probably because ifenprodil prevented memory destabilization. NR2B-NMDARs interact with nitric oxide synthase (NOS)^33^,^34^, which is implicated in the epigenetic regulation of Arc^35^. Therefore, NR2B-NMDAR activation could contribute to late Arc expression through epigenetic priming of *Arc* transcription.

A possible mechanism by which late Arc expression contribute to memory persistence is refinement of neuronal circuit through pruning of dendritic spines. We previously have shown that persistence of newly-acquired memory is associated with late Arc-dependent pruning of small mushroom spines in hippocampal CA1 neurons 7 d following fear conditioning^20^. Because the strengthening of specific synaptic connections underlies a memory trace, elimination of redundant synapses could refine functional circuits for memory. Although it remains unknown whether spine pruning in the BLA is following memory retrieval, the similar synaptic mechanisms to newly-acquired memory could be involved in persistence of reactivated memory. Stabilization of 7-d but not 2-d new and reactivated memories may require late Arc-dependent spine pruning.

The different roles of the lateral (LA) and basal amygdala (BA) are of interest. Classically, the BA is thought to be implicated in contextual fear and the LA in cued fear. However, contextual fear is also encoded in the LA^36^,^37^. In this study, we did not distinguish these areas. Further studies are helpful for identifying more specific role of the LA and BA.

In conclusion, we report that late Arc expression is essential for the persistence of newly-acquired and reactivated memories. Persistent fear memories could be a factor in post-traumatic stress disorder^1^. Therefore, pharmacological manipulation of delayed cellular signaling following initial learning and memory retrieval could be a possible therapeutic intervention for these disorders.

## Materials and Methods

### Mice

All experiments performed were approved by the animal experiment ethics committee at the University of Tokyo (approval number 24–10), and were in accordance with the University of Tokyo guidelines for the care and use of laboratory animals. Adult male C57BL/6 J mice (Japan SLC Inc., Shizuoka, Japan), weighing 20–30 g and aged 8–13 weeks, were housed 2–4 per cage, and kept on a 12-h light/dark cycle (lights on from 7 a.m. to 7 p.m.). All mice were given free access to food and water, and acclimated to daily handling for 1 week prior to the start of the study.

### Behavioral procedures

Contextual fear conditioning and subsequent testing were performed in a conditioning chamber (18 cm wide, 15 cm deep and 27 cm high) that had a stainless steel grid floor^38^. The chamber was cleaned with 70% ethanol before each session. A conditioning session consisted of placing the mice in the chamber and delivering a 2-s footshock (1 mA) after 148 s. The mice then received 2 additional shocks every 148 s. They were kept in the chamber for an additional 60 s and were then returned to their home cages. An immediate shock session consisted of delivering 2-s footshocks 3 times to the mice immediately after placing them in the chamber. The mice then remained in the chamber for 500 s. The retrieval and testing sessions consisted of exposing the mice to the conditioning chamber for 5 min, in the absence of a footshock. We employed 5 min of exposure, since we preliminary confirmed that 5 min of exposure reliably induced memory destabilization. In the reminder shock session, mice received mild footshock (0.6 mA, 2 s) in a neutral chamber (triangle, 21 × 21 cm and 26 cm) immediately after being placed in the chamber. The mice were then returned to their home cages immediately after receiving footshock. We previously confirmed that this mild immediate shock reinstates extinguished fear^25^. All sessions were performed between 8 a.m. and 12 p.m., and each session was video-recorded for automatic scoring of freezing, according to a previously described method^39^. Images were captured at two frames per second. When the amount of area within the mouse moved was below a certain threshold for more than 1 s, behavior was judged as ‘freezing.’ The optimal threshold by which we judged freezing was determined by adjusting it to the amount of freezing measured by human observation. In the human observation, freezing was defined as the absence of all movements, except those related to breathing.

### Western blotting

Mice were decapitated after diethyl- ether anesthesia, and their brains were rapidly removed and frozen at −80 °C. Coronal brain sections (300 μm) were prepared using a cryostat (HM520; Thermo Fisher Scientific, Waltham, MA, USA). The basolateral amygdala (0.90 to 2.00 mm posterior to bregma) was punched out and homogenized in RIPA buffer (08714-04, Nacalai Tesque, Kyoto, Japan). Protein concentrations were normalized across homogenates using the BCA method (Thermo Scientific). Equal amounts of protein were electrophoresed on 5–20% SDS polyacrylamide gels and transferred to nitrocellulose membranes. Western blots were blocked in blocking buffer (03953-95, Nacalai Tesque, JAPAN) and then incubated with an anti-Arc/Arg3.1 antibody (sc-17839, Santa Cruz Biotechnology) at a 1:1000 dilution or with an anti-β-actin antibody (A3854, Sigma) at a 1:10,000 dilution. After incubation with anti-Mouse IgG (A9044, Sigma) at a 1:100,000 dilution, bands were developed with a chemiluminescent substrate (RPN2132, GE Healthcare). The immunopositive signals were quantified by ImageQuant LAS 4000 (GE Healthcare). Arc/Arg3.1 signals were normalized with β-actin signals.

### Fluorescent *in situ* hybridization

After the mice were sacrificed, the brains were quick-frozen and stored at −80 °C until further processing. Coronal brain sections (20 μm) were prepared using a cryostat and mounted on slides. Slides were air-dried and stored at −80 °C until use. Regions containing amygdala were selected for *in situ* hybridization. Antisense riboprobes for *Arc*, conjugated to digoxigenin-UTP (Roche Applied Science, Penzberg, Germany), were generated from cDNA plasmid (provided by Dr. Paul Worley) containing an almost full-length cDNA of the *Arc* transcript using MAXIScript (Ambion, Austin, TX USA). *In situ* hybridization was performed as previously published protocols^40^. Slide-mounted brain sections were fixed in 4% buffered paraformaldehyde, acetylated with 0.5% acetic anhydride/1.5% triethanolamine, dehydrated through 50% methanol/50% acetone solutions, and equilibrated in 2 × saline sodium citrate buffer (SSC). Slides were incubated in prehybridization buffer for 30 min. The antisense riboprobe was diluted to 150 μl in hybridization buffer, heat denatured, chilled on ice, and applied to each slide. Hybridization was carried out at 56 °C for 16 h. Slides were washed to a final stringency of 0.5 × SSC at 56 °C, which included an earlier wash step at 37 °C in 2 × SSC with RNase A (10 μg/ml). Endogenous peroxidase activity was quenched with 2% H_2_O_2_ in 1 × SSC. Slides were blocked with TSA blocking reagent (PerkinElmer, Waltham, MA USA) and incubated with an anti-digoxigenin horseradish peroxidase (HRP)-conjugate (1:500, Roche) for 2 h. Slides were washed 3 times in Tris-buffered saline with 0.05% Tween-20, and the conjugate was detected using biotin-labeled tyramide and Alexa Fluor 488 streptavidin (0.5 μg/mL; Life Technologies, Carlsbad, CA USA)^41^. Slides were washed in PBS, and the nuclei were counterstained with propidium iodide (PI, 10 μM, Life Technologies) for 10 min. Finally, the sections were washed with PBS and mounted in Permafluor (Thermo Fisher Scientific, Waltham, MA USA).

### Immunohistochemistry

After anesthesia with diethyl ether, mice were transcardially perfused with phosphate buffered saline (PBS) followed by 4% paraformaldehyde (PFA). Brains were post-fixed in 4% PFA for 12 h. Free-floating coronal sections (40 μm) were prepared using a cryostat. Arc was visualized with an anti-Arc/Arg3.1 primary antibody (156 003, 1:1,000; Synaptic Systems, Gottingen, Germany), biotinylated anti-rabbit secondary antibody (BA-1000, 1:500; Vector Laboratories, Burlingame, CA, USA), VECTASTAIN ABC Kit (Vector Laboratories), and TSA Plus Cyanine 3 System (NEL744001KT, 1:100; Perkin-Elmer, Waltham, MA, USA). Nuclei were counterstained with Hoechst dye (1:1,000; Life Technologies).

### Confocal microscopy and image analysis following immunohistochemistry

Images of BLA neurons (1.00 to 1.50 mm posterior to bregma, 4–6 slices per mouse) were acquired using a confocal microscope (CV1000; YOKOGAWA, Tokyo, Japan) at 40 × under an oil-immersion lens (NA, 1.3). Areas of analysis were z-sectioned in 1.6-μm optical sections. The same laser and scanning settings were used for images within an experiment to allow for comparison across groups. Fluorescence images were analyzed using ImageJ software (NIH). The nuclei in the BLA area were traced in the Hoechst channel using ImageJ software. Only cells that were presumptive neurons, with large nuclei stained diffusely with Hoechst, were included in the analysis. The designation “Arc positive (Arc+)” was assigned to cells containing perinuclear labeling over three optical sections.

### Confocal microscopy and image analysis following *in situ* hybridization

Images were collected using a confocal microscope (LSM 510; Carl Zeiss AG, Oberkochen, Germany) at 40× under a water-immersion lens (NA 1.2). Photomultiplier tube assignments and pinhole size were kept constant, while laser power and gain were optimized on each slide to detect relatively weaker cytoplasmic *Arc* signals. Images of the basolateral amygdala were acquired by collecting z-stacks (1-μm-thick optical sections). Images were analyzed on the MetaMorph 6.0 program (Molecular Devices, LLC, Sunnyvale, CA USA). Only cells containing whole nuclei were included in the analysis. The PI stains revealed nuclei of 2 distinct morphologies. The majority of nuclei in the amygdala were large and stained diffusely with PI. Only these presumptive neurons were included in the analysis. The remainder of the cells, presumably glial cells, had much smaller nuclei, stained strongly with PI, and did not express *Arc* mRNA. The designation “*Arc*+” was given to cells containing perinuclear/cytoplasmic labeling over more than 3 optical sections.

### Surgery

Mice were anesthetized with pentobarbital (2.5 mg/kg, i.p.) and xylazine (10 mg/kg, i.p.), and 26-gauge stainless-steel guide cannulas (Plastics One) were implanted in the amygdala (−1.3, ±3.1, −4.6 mm relative to bregma). Cannulas were secured to the skull using a mixture of acrylic and dental cement, and 33-gauge dummy cannulas were then inserted into each guide cannula to prevent clogging. Mice were given at least 7 d of postoperative recovery time.

### Oligodeoxynucleotide design

*Arc/Arg3.1* antisense ODN and scrambled ODN were designed in reference to a previous study^17^. The *Arc/Arg3.1* ODN encoded an antisense sequence for the *Arc/Arg3.1* mRNA sequence near the translation start site^42^. The scrambled ODN does not show significant homology to any sequences in the GenBank database^17^. Both ODNs contained phosphorothioate linkages on the three terminal bases of both the 5′ and 3′ ends and phosphodiester internal bonds. This design is reported as more stable than unmodified phosphorothioated ODNs *in vivo* and less toxic than fully phosphorothioated ODNs^17^. The following sequences were used (“~” denotes a phosphorothioate linkage): 5′-G~T~C~CAGCTCCATCTGGT~C~G~T-3′ (antisense) and 5′-C~G~T~GCACCTCTCGCAGG~T~T~T-3′ (scrambled). This antisense sequence has been shown to effectively inhibit Arc/Arg3.1 protein expression in the hippocampus and to exhibit a high degree of specificity for Arc relative to other immediate early genes^17^,^23^ .

### Drugs and microinfusions

Mice received intra-amygdala infusion (0.5 μL per side) of ODN (400 pmol), threo ifenprodil hemitartrate (2.5 μg, Tocris Bioscience), anisomycin (62.5 μg, Sigma) or NVP-AAM077 (0.2 μg, Sigma). The volume of drugs that we used spreads within only the BLA in previous studies^43^,^44^. The solutions were dissolved in PBS or 100 mM DMSO (WAKO, JAPAN), and PBS, DMSO was used as vehicle solution. Infusions were made over 2 min, and the infusion cannulas (28 gauge, extending 0.5 mm below the guide cannula) were left in place for at least 2 min afterwards in order to facilitate the diffusion of solutions throughout the amygdala.

### Data analysis

All values were reported as means ± SEMs. Statistical analysis was performed using two-tailed Student’s *t*-test, paired *t*-test, one-way analysis of variance (ANOVA), repeated-measures ANOVA and Tukey’s test, where appropriate. *P* < 0.05 is considered statistically significant. Statistical results were shown in Supplementary Table 1.

## Additional Information

**How to cite this article**: Nakayama, D. *et al.* Late Arc/Arg3.1 expression in the basolateral amygdala is essential for persistence of newly-acquired and reactivated contextual fear memories. *Sci. Rep.* **6**, 21007; doi: 10.1038/srep21007 (2016).

## Supplementary Material

Supplementary Information
