# Supplementary material for: Late Arc/Arg3.1 expression in the basolateral amygdala is essential for persistence of newly-acquired and reactivated contextual fear memories
Source: Sci Rep. 2016 Feb 16;6:21007. doi: 10.1038/srep21007 (PMC4754630; doi:10.1038/srep21007)
Supplement: Supplementary Information [file srep21007-s1.pdf]

Supplementary information

Late Arc/Arg3.1 expression in the basolateral amygdala is essential for persistence of newly-acquired and reactivated contextual fear memories

Daisuke Nakayama, Yoshiko Hashikawa-Yamasaki, Yuji Ikegaya
, Norio Matsuki and Hiroshi Nomura*

Supplementary figures

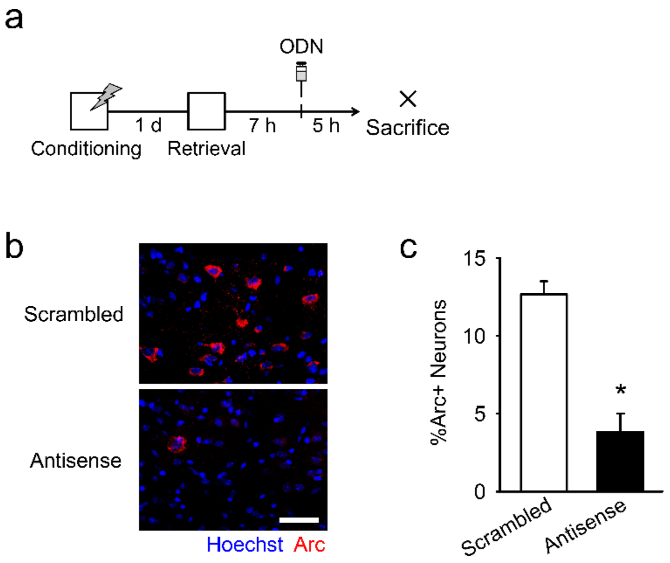

Supplementary Figure S1

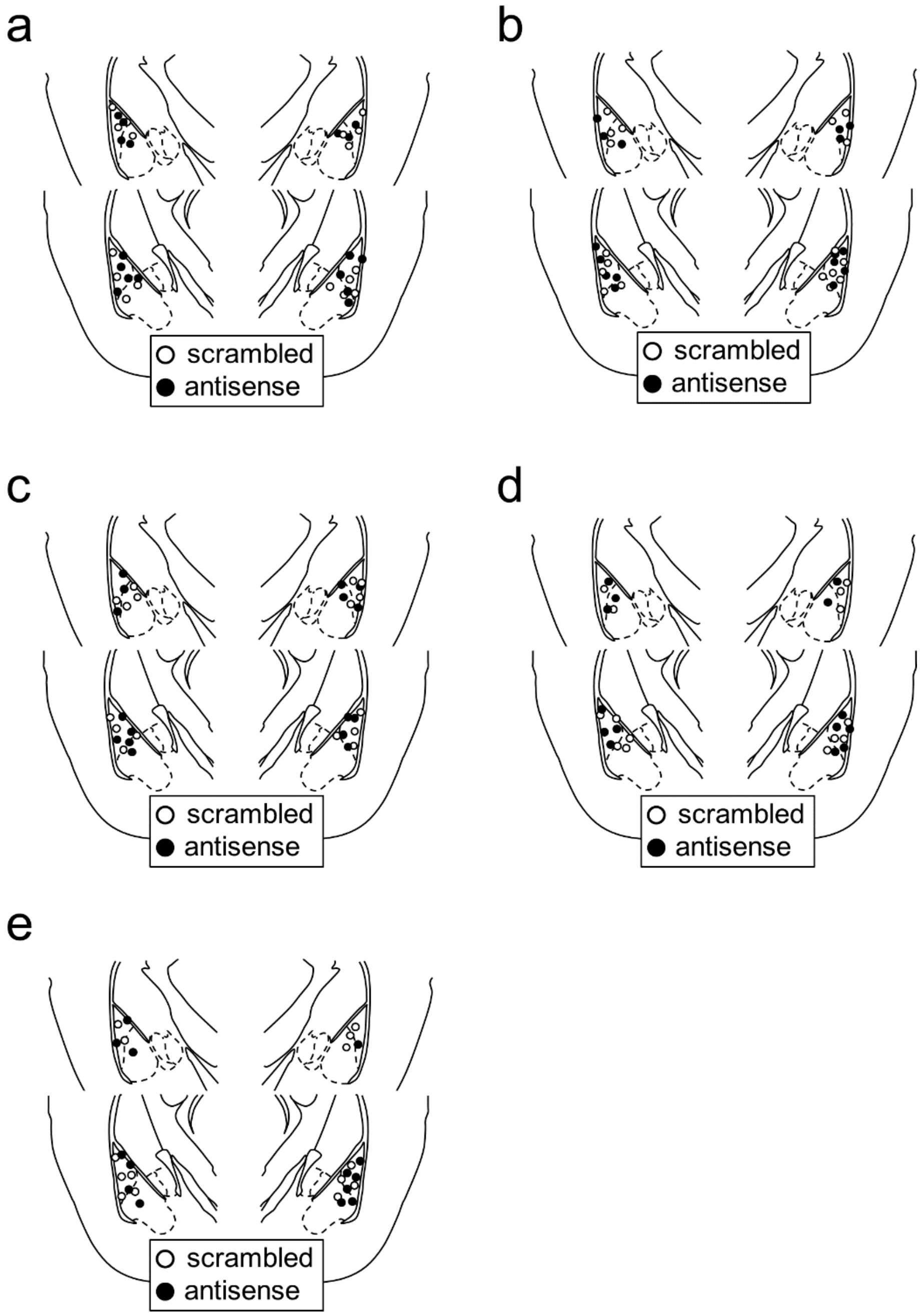

Supplementary Figure S2

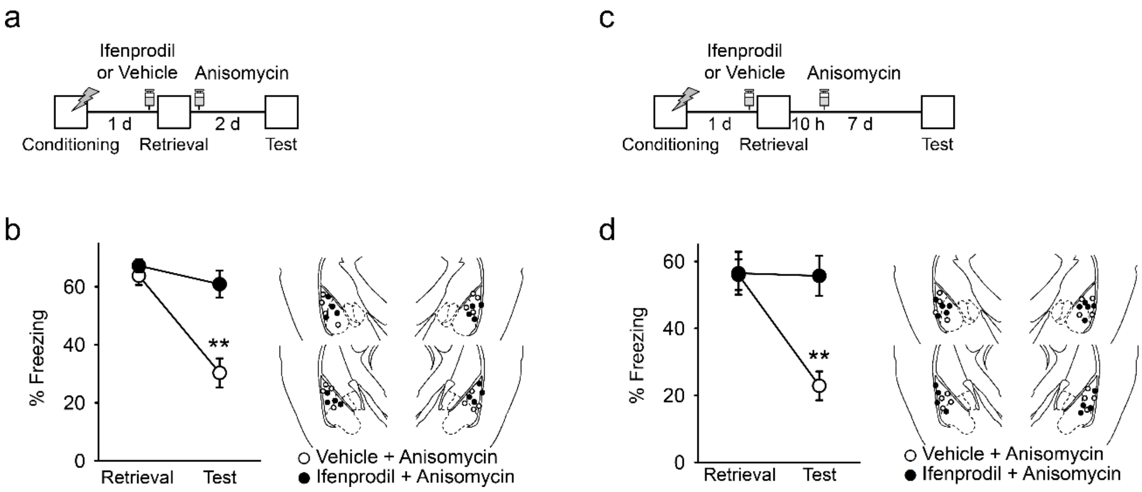

Supplementary Figure S3

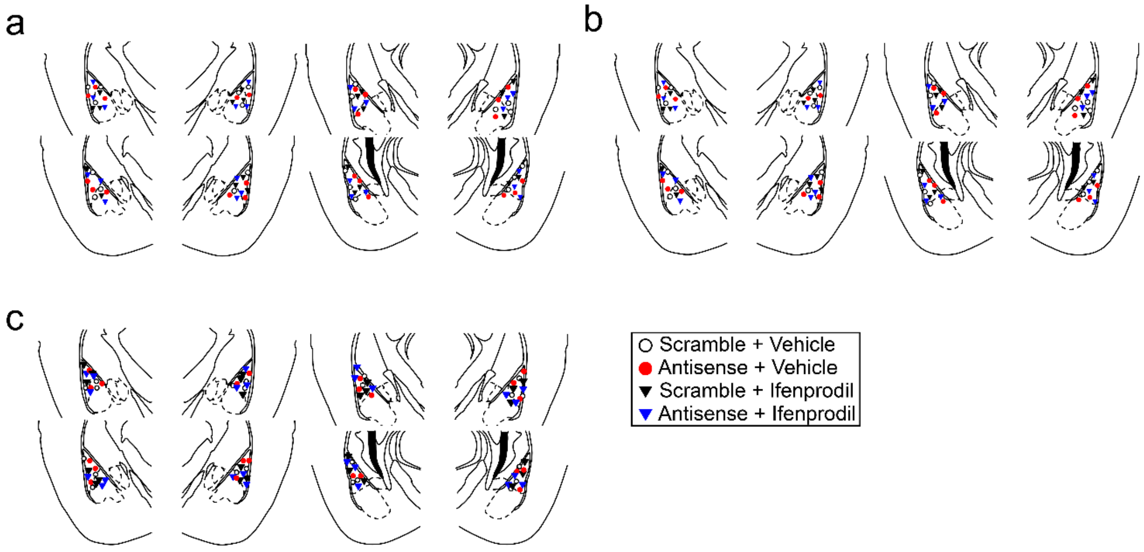

Supplementary Figure S4

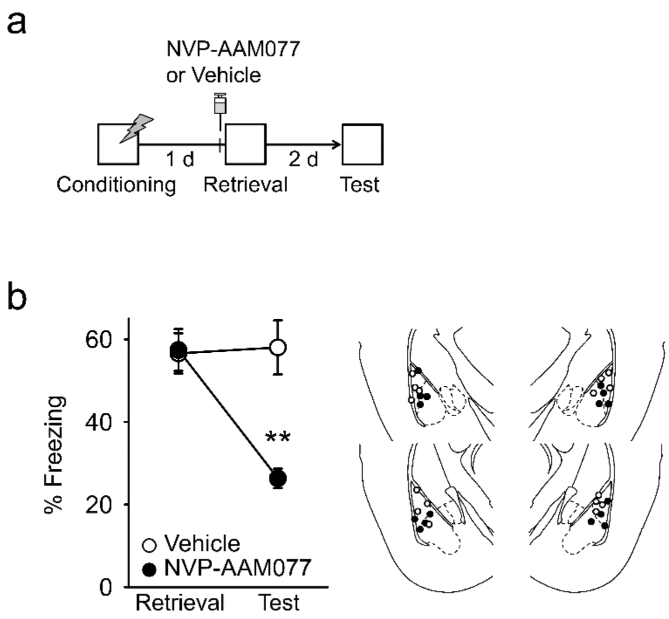

Supplementary figure S5

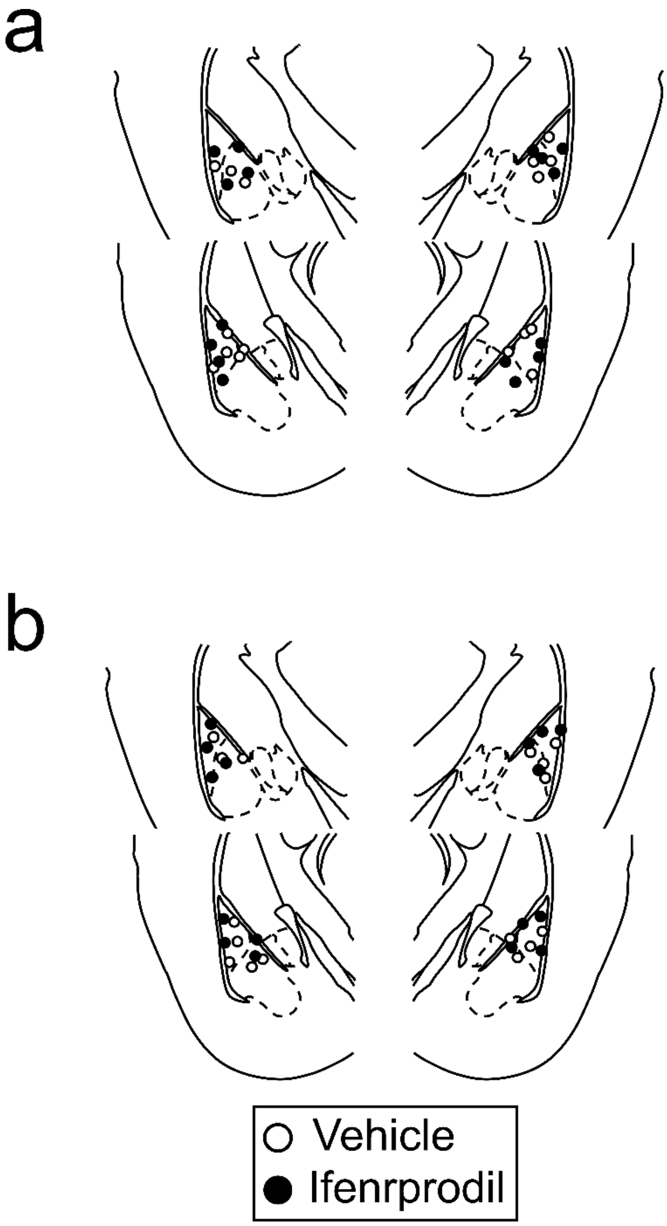

Supplementary figure S6

Supplementary Table

Supplementary Table 1 Statistical results

**Figure**	**Statistical tests and results**
Figure 1A	One-way ANOVA, $F_{(4, 45)}$ = 6.8, *P* = 0.00023; Tukey's post hoc test, Naïve vs. 2 h, ***P* = 0.0043; 12 h, **P* = 0.012
Figure 1B	One-way ANOVA, $F_{(4, 45)}$ = 8.1, *P* = 5.1× 10^{-5} ; Tukey's post hoc test, Naïve vs. 2 h, ***P* = 0.0044; 12 h, ***P* = 0.00067
Figure 1D	Student's *t*-test, $t_{(24)}$ = 7.1, ***P* = 2.5 × 10^{-7}
Figure 1E	Student's *t*-test, $t_{(14)}$ = 0.22, *P* = 0.83<br>One-way ANOVA, $F_{(2, 36)}$ = 5.58, *P* = 0.0077; Tukey's post hoc test, IS vs. FC, **P* = 0.029
Figure 1H	One-way ANOVA, $F_{(2,12)}$ = 54.9, P = 9.2 ×10^{-7}
Figure 2B	(Left) Student's *t*-test, $t_{(16)}$ = 0.19, *P* = 0.85<br>(Right) Student's *t*-test, $t_{(15)}$ = 5.1, ***P* = 0.00013
Figure 2D	(Left) Repeated measures ANOVA, $F_{(1,14)}$ = 0.25, *P* = 0.63<br>(Right) Repeated measures ANOVA, $F_{(1,12)}$ = 8.4, *P* = 0.013; Scrambled vs. Antisense in test session, ***P* = 0.0086<br>Paired t-test, $t_{(6)}$ = 0.81, *P* = 0.45
Figure 3B	Repeated measures ANOVA, $F_{(3,41)}$ = 15.3, *P* = 8.1 × 10^{-7}; Tukey's post-hoc test, Scrambled + Vehicle vs. Antisense + Vehicle in test session, ***P* = 6.6 × 10^{-5}; Antisense + Vehicle vs. Antisense + Ifenprodil in test session, ***P* = 7.3 × 10^{-5}
Figure 3D	Repeated measures ANOVA, $F_{(3,41)}$ = 12.0, P = 9.1 × 10^{-6}; Tukey's post-hoc test, Scrambled + Vehicle vs. Antisense + Vehicle in test session, ***P* =0.0041; Antisense + Vehicle vs. Antisense + Ifenprodil in test session, **P* = 0.013
Figure 3F	Repeated measures ANOVA, $F_{(3,42)}$ = 10.4, *P* = 3.0 × 10^{-5}; Tukey's post-hoc test, Scrambled + Vehicle vs. Antisense + Vehicle in test session, P = 5.0 × 10^{-4}; Antisense + Vehicle vs. Antisense + Ifenprodil in test session, P = 0.99
Figure 4B	one-way ANOVA, $F_{(2, 21)}$ = 7.2, *P* = 0.0042; Tukey's post hoc test, Vehicle vs Ifenprodil, **P* = 0.026
Figure 4D	one-way ANOVA, $F_{(2, 21)}$ = 5.7, *P* = 0.010; Tukey's post hoc test, Vehicle vs Ifenprodil, **P* = 0.038

Supplementary figure legends

Supplementary Figure S1 Infusions of *Arc* antisense decrease proportion of Arc^+ neurons in the BLA, related to Figure 2.

(A) Mice were infused with Arc antisense or scrambled ODNs into the BLA 7 h after memory retrieval and sacrificed 5 later (n = 4 mice). (B) Representative images of Arc immunostaining in the BLA. Scale bar, 50 μm. (C) Infusions of *Arc* antisense decreased proportion of Arc+ neurons in the BLA (Student's *t*-test, $t_{(4)} = 4.5$, $P = 0.011$).

Supplementary Figure S2 Histological verification of cannula placements, related to Figure 2.

(A) Histological verification of cannula placements, related to Figure 2B left. (B) Histological verification of cannula placements, related to Figure 2B right. (C) Histological verification of cannula placements, related to Figure 2D left. (D) Histological verification of cannula placements related to Fig. 2D right. (E) Histological verification of cannula placements related to Figure 2F.

Supplementary Figure S3 NR2B-NMDARs are involved in memory destabilization that entails early and late protein expression

(A) Mice received intra-BLA injections of ifenprodil or vehicle 5 min before memory retrieval and anisomycin after memory retrieval (Vehicle + Anisomycin: n = 8 mice, Ifenprodil + Anisomycin: n =

8 mice). (B) (Left) The ifenprodil injections prevented memory impairment resulting from inhibiting early protein expression (repeated measures ANOVA, $F_{(1, 14)}$ = 25.3, P = 0.00018; Vehicle + Anisomycin vs. Ifenprodil + Anisomycin in test session, **P = 0.00051). (Right) Histological verification of cannula placements expression. (C) Mice received intra-BLA injections of ifenprodil or vehicle 5 min before memory retrieval and anisomycin 10 h after memory retrieval (Vehicle + Anisomycin: n = 9 mice, Ifenprodil + Anisomycin: n = 8 mice). (D) (Left) The ifenprodil injections prevented memory impairment resulting from inhibiting late protein expression (repeated measures ANOVA, $F_{(1, 15)}$ = 11.8, P = 0.0037; Vehicle + Anisomycin vs. Ifenprodil + Anisomycin in test session, **P = 0.00038). (Right) Histological verification of cannula placements.

Supplementary Figure S4 Histological verification of cannula placements, related to Figure 3.

(A) Histological verification of cannula placements, related to Fiture 3B. (B) Histological verification of cannula placements, related to Figure 3D. (C) Histological verification of cannula placements related to Figure 3F.

Supplementary Figure S5 Infusions of NR2A-preferring NMDAR antagonist disrupts memory reconsolidation

(A) Mice were infused with NVP-AAM077 into the BLA 5 min before memory retrieval. (B) (Left)

NVP-AAM077 treatment disrupted freezing behavior in the 2 d test (repeated measures ANOVA, $F_{(1, 14)}$ = 15.7, *P* = 0.0014; Vehicle vs. NVP-AAM077 in test session, ***P* = 0.00045). (Right) Histological verification of cannula placements.

Supplementary Figure S6 Histological verification of cannula placements, related to Figure 4.

(A) Histological verification of cannula placements, related to Figure 4B. (B) Histological verification of cannula placements related to Figure 4D.
